# Supplementary material for: Heat adaptation of phage T7 under an extended genetic code
Source: Virus Evol. 2021 Dec 1;7(2):veab100. doi: 10.1093/ve/veab100 (PMC8923235; doi:10.1093/ve/veab100)
Supplement: veab100_Supp [file veab100_supp.zip › Sequencing_Analysis.zip!/Sequencing_Analysis/A2/evidence/MC_SIDE_2_40.html]

BRESEQ :: Evidence


| Predicted mutation | | | | | | |
| --- | --- | --- | --- | --- | --- | --- |
| evidence | seq id | position | mutation | annotation | gene | description |
| MC JC | NC\_001604.1 | 1,257 | Δ1,480 bp | intergenic (–/–) | – / – | –/– |

| Missing coverage evidence... | | | | | | | | | | |
| --- | --- | --- | --- | --- | --- | --- | --- | --- | --- | --- |
|  |  |  | seq id | start | end | size | ←reads | reads→ | gene | description |
| \* | \* | ÷ | NC\_001604.1 | 1257 | 2736 | 1480 | 473 [0] | [0] 475 | *–/–* | –/– |

| New junction evidence | | | | | | | | | | | |
| --- | --- | --- | --- | --- | --- | --- | --- | --- | --- | --- | --- |
|  | | seq id | position | reads (cov) | reads (cov) | score | skew | freq | annotation | gene | product |
| \* | ? | NC\_001604.1 | = 1256 | 0 (0.000) | 452 (1.040) | 181/574 | 0.1 | 100% | intergenic (–/–) | *–/–* | –/– |
|  | ? | NC\_001604.1 | 2737 = | 0 (0.000) |  |  |  |  | intergenic (–/–) | *–/–* | –/– |

Only 100 of 475 total aligned reads displayed.  

`TGCGAGCGTTTCAACAATGATGCCCATTATAAATACGCTGAGATTGCAAGCGACATCATTGATTGCAATTCGGATGAGCATGATGAGTTAACTGGATGGGATGGTGAGTTTGTTGAAACTTGTAAACTAATCCGCAAGTTCTTTGAGGGCATCGCCTCATTCGACATGCATAGCGGGAACATCATGTTCTCAAATGGAGACGTACCATACATCACCGACCCGGTATCATTCTCGCAGAAGAAAGACGGTGGCGCATTCAGCATCGACCCTGAGGAACTCATCAAGGAAGTCGAGGAAGTCGCACGACAGAAAGAAATTGACCGCGCTAAGGCCCGTAAAGAACGTCACGAGGGGCGCTTAGAGGCACGCAGATTCAAACGTCGCAACCGCAAGGCACGTAAAGCACACAAAGCTAAGCGCGAAAGAATGCTTGCTGCGTGGCGATGGGCTGAACGTCAAGAACGGCGTAACCATGAGGTAGCTGTAGATGTACTAGGAAGAACCAATAACGCTATGCTCTGGGTCAACATGTTCTCTGGGGACTTTAAGGCGCTTGAGGAACGAATCGCGCTGCACTGGCGTAATGCTGACCGGATG  >  NC_001604.1/2441‑3037`  

                                                                                                                                                                                                                                                                                                        `|`                                                                                                                                                                                                                                                                                                              
`caccacgcttacgaaatgctgaaacaaaacatccgttatgatgacatccgtgacactgatgacctgcacgatgctattcacatggctgccgataatgcagttccgcactactacgctgacatctttagcgtaatggcaagtgagggcattgaccttgagttcgaagactctggtctgatgcctgacaccaaggacgtaatccgcatcctgcaagcgcgtatctatgagcaattaacgattgacctctgggaagacgcagaagacttgctcaatgaatacttggaggaagtcgaggaagtcg                                                                                                                                                                                                                                                                                                          <  1:41677‑M2/5‑1 (MQ=255)`  
     `cgcttacgaaatgctgaaagaaaacatccgttatgatgacatccgtgacactgatgacctgcacgatgctattcacatggctgccgataatgcagttccgcactactacgctgacatctttagcgtaatggcaagttagggcattgaccttgagttcgaagactctggtctgatgcctgacaccaaggacgtaatccgcatcctgcaagcgcgtatctatgagcaattaacgattgacctctgggaagacgcagaagacttgctcaatgaatacttggaggaagtcgaggaAGTCGCAcgc                                                                                                                                                                                                                                                                                                     >  1:635298‑M2/292‑300 (MQ=255)`  
                    `gaaagaaaacatccgttatgatgacatacgtgacactgatgacctgcacgatgctattcacatggctgccgataatgcagttccgcactactacgctgacatctttagcgtaatggcaagtgagggcattgaccttgagttcgaagactctggtctgatgcctgacaccaaggacgtaatccgcatcatgcaagcgcgtatctatgagcaattaacgattgacctctgggaagacgcagaagacttgctcaatgaatacttggaggaagtagaggaAGTCGCACGACAGAAAGAAATTGAc                                                                                                                                                                                                                                                                                      >  1:86414‑M2/277‑301 (MQ=255)`  
                               `cccgttatgatgacatccgtgacactgatgacctgcacgatgctattcacatggctgccgataatgcagttccgcactactacgctgacatctttagcgtaatggcaagtgagggcattgaccttgagttcgaagactctggtctgatgcctgacaccaaggacgtaatccgcatcctgcaagcgcgtatctatgagcaattaacgattgacctctgggaagacgcagaagacttgctcaatgaatacttggaggaagtcgaggaAGTCGCACGACAGAAAGAAATTGACCGCGCTAAGGc                                                                                                                                                                                                                                                                           <  1:507863‑M2/36‑1 (MQ=255)`  
                                      `tgatgacatccgtgacactgatgacctgcacgatgctattcacatggctgccgataatgcagttccgcactactacgctgacatctttagcgtaatggcaagtgagggcattgaccttgagttcgaagactctggtctgatgcctgacaccaaggacgtaatccgcatcctgcaagcgcgtatctatgagcaattaacgattgacctctgggaagacgcagaagacttgctcaattaatacttggaggaagtcgaggaAGTCGCACGACAGAAAGAAATTGACCGCgctaaggctgtctct                                                                                                                                                                                                                                                                    >  1:578829‑M2/259‑294 (MQ=255)`  
                                       `gatgacatccgtgacactgatgacctgcacgatgctattcacatggctgccgataatgcagttccgcactactacgctgacatctttagcgtaatggcaagtgagggcattgaccttgagttcgaagactctggtctgatgcctgacaccaaggacgtaatccgcatcctgcaagcgcgtatctatgagcaattaacgattgacctctgggaagacgcagaagacttgctcaatgaatacttggaggaagtcgaggaAGTCGCACGACAGAAAGAAATTGACCGCGCTAAGGCCCGTAAAg                                                                                                                                                                                                                                                                   <  1:697410‑M2/44‑1 (MQ=255)`  
                                       `gataacatccgtgacactgatgacctgcacgatgctattcacatggctgccgataatgcagttccgcactactacgctgacatctttagcgtaatggcaagtgagggcattgaccttgagttcgaagactctggtctgatgcctgacaccaaggacgtaatccgcatcctgcaagcgcgtatctatgagcaattaacgattgacctctgggaagacgcagaagacttgctcaatgaatacttggaggaagtcgaggaAGTCGCACGACAGAAAGAAATTGACCGCGCTAAGGCCCGTAAAg                                                                                                                                                                                                                                                                   >  1:619489‑M2/258‑301 (MQ=255)`  
                                           `gttatgatgacatccgtgacactgatgacctgcacgatgctattcacatggctgccgataatgcagttccgcactactacgctgacatctttagcgtaatggcaagtgatggcattgaccttgagttcgaagactctggtctgatgcctgacaccaaggacgtaatccgcatcctgcaagcgcgtatctatgagcaattaacgattgacctctgggaagacgcagaagacttgctcaatgactacttggaggaAGTCGCACGACGGAAAGAAATTGACCGCGCTAAGgcccgtaactgtct                                                                                                                                                                                                                                                               >  1:32509‑M2/254‑295 (MQ=255)`  
                                             `atccgtgacactgatgacctgcacgatgctattcacatggctgccgataatgcagttccgcactactacgctgacatctttagcgtaatggcaagtgagggcattgaccttgagttcgaagactctggtctgatgcctgacaccaaggacgtaatccgcatcctgcaagcgcgtatctatgagcaattaacgattgacctctgggaagacgcagaagacttgctcaatgaatacttggaggaagtcgaggaAGTCGCACGACAGAAAGAAATTGACCGCGCTAAGGCACGTAAAGAACGTc                                                                                                                                                                                                                                                             >  1:90316‑M2/252‑301 (MQ=255)`  
                                                `cgtgacactgatgacctgcacgatgctattcacatggctgccgataatgcagttccgcactactacgctgacatctttagcgtaatggcaagtgagggcattgaccttgagttcgaagactctggtctgatgcctgacaccaaggacgtaatccgcatcctgcaagcgcgtatctatgagcaattaacgattgacctctgggaagacgcagaagacttgctcaatgaatacttggaggaagtcgaggaAGTCGCACGACAGAAAGAAATTGACCGCGCTAAGGCCCGTAAAGAACGTCACg                                                                                                                                                                                                                                                          <  1:746175‑M2/53‑1 (MQ=255)`  
                                                      `actgatgacctgcacgatgctattcacatggctgccgataatgcagttccgcactactacgctgacatctttagcgtaatggcaagtgagggcattgaccttgagttcgaagactctggtctgatgcctgacaccaaggacgtaatccgcatcctgcaagcgcgtatctatgagcaattaacgattgacctctgggaagacgcagaagacttgctcaatgaatacttggaggaagtcgaggaAGTCGCACGACAGAAAGAAATTGACCGCGCTAAGGCCCGTAAAGAACGTCACGaggggg                                                                                                                                                                                                                                                    >  1:539928‑M2/243‑300 (MQ=255)`  
                                                        `cagggacactgatgacctgcacgatgcgattcacatggctgccgataatgcagttccgcactactacgctgacatctttagcgtaatggcaagtgagggcattgaccttgagttcgaagactctggtctgatgcctgacaccaaggacgtaatccgcatcctgcaagcgcgtatctatgagcaattaacgatttacctctgggaagacgcagaagacttgctcaatgaatactttgaggaAGTCGCACGACAGAAATAAATTGACCGCGCTAAGGCCCGTAAAGAACGTCACGAGGGgcgc                                                                                                                                                                                                                                                  <  1:704478‑M2/61‑1 (MQ=255)`  
                                                                    `ggatgctattcacatggctgccgataatgcagttccgaactactacgctgacatctttagcgtaatggcaagtgagggcattgaccttgagttcgaagactctggtctgatgcctgacaccaaggacgtaagccgcatcctgcaagcgcgtatctatgagcaattaacgattgacctctgggaagacgcagaagacttgctaaatgaatactgggaggaagtcgaggaAGTCGCACGACAGAAAGAAATTGACCGCGCTAAGGCCCGTAAAGAACGTCACGAGGGGCGCTTAGAGGCACGc                                                                                                                                                                                                                                      <  1:448267‑M2/73‑1 (MQ=255)`  
                                                                         `ctattcacatggctgccgataatgcagttccgcactactacgctgacatctttagcgtaatggcaagtgagggcattgaccttgagttcgaagactctggtctgatgcctgacaccaaggacgtaatccgcatcctgcaagcgcgtatctatgagcaattaacgattgacctctgggaagacgcagaagacttgctcaatgaatacttggaggaagtcgacgaAGTCGCACGACAGAAAGAAATTGACCGCGCTAAGGCCCGTaaagaacgtcccgaggggcgcctgtctcttatacacat                                                                                                                                                                                                                                 >  1:630253‑M2/224‑284 (MQ=255)`  
                                                                             `tcacatggctgccgataatgcagttccgcactactacgctgacatctttagcgtaatggcaagtgaggccattgaccttgagttcgaagactctggtctgatgcctgacaccaaggacgtaatccgcatcctgcaagcgcgtatctatgagcaattaacgattgacctctgggaagacgcagaagacttgctcaatgaatacttggaggaagtcgagtaAGTCGCACGACAGCAAGCAATTGAccgcgctaaggcccgtaaagaacgtcaagagctgtctcttatacacatctccgagccc                                                                                                                                                                                                                             >  1:563558‑M2/220‑274 (MQ=255)`  
                                                                              `cacatggctgccgataatgcagttccgcactactacgctgacatctttagcgtaatggcaagtgagggcattgaccttgagttcgaagactctggtctgatgcctgacaccaaggacgtaatccgcatcctgcaagcgcgtatctaagagcaattaacgattgacctctgggaagacgcagaagacttgctcaatgaatacttggaggaagtcgaggaAGTCGCCCGACAGAAAGAAATTGACCGCGCTAAGGCCCGTAAAGAACGTCACGAGGGGCGCTTAGAGGCACGCAGATTCAAAc                                                                                                                                                                                                                            <  1:740586‑M2/83‑1 (MQ=255)`  
                                                                              `cacatggctgccgataaggcagttccgcactactacgctgacatctttagcgtaatggcaagtgagggcattgaccttgagttcgaagactctggtctgatgcctgacaccaaggacgtaatccgcatcctgcaagcgcgtatctatgagcaattaacgattgacctctgtgaagacgcagaagacttgctcaatgaatacttggaggaagtcgaggaAGTCGCACGACAGAAAGAAATTGACCGCGCTAAGGCCCGTAAAGAACGTCACTAGGGGCGCTTAGAGGCACGCAGATTCAAAc                                                                                                                                                                                                                            <  1:810463‑M2/83‑1 (MQ=255)`  
                                                                              `cacatggctgccaataatgcagttccgcacaactacgctgacatctttatcgtaatggcaagtgagggcattgacctggagttcgaagactctggtctgatgcctgacaccaaggacgtaatccgcatcctgcaagcgcgtatctatgagcaattaacgattgacctctgggaagacgcagaagacttgctcaatgaatacttggaggaagtcgaggaAGTCGCACGACAGAAAGAAATTGACCGCGCTAAGGCCCGTAAAGAACGTCACGAGGGGCGCTTAGAGGCACGCAGATTCAAAc                                                                                                                                                                                                                            <  1:42640‑M2/83‑1 (MQ=255)`  
                                                                                    `gctgccgataatgcagttccgcactactatgctgacatctttagcgtaatggcaagtgagggcattgaccttgagttcgaagactctggtctgatgcctgacaccaaggacgtaatccgcatcctgcaagcgcgtatctatgagcaattaacgattgacctctgggaagacgaagaagacttgctcaatgaatacttggaggaagtcgaggaAGTCGCACGACAGAAAGAAATTGACCGCGCTAAGGCCCGTAAAGAACGTCACGAGGGGCGCTTAGAGGCACGCAGATTCAAACGTCGCa                                                                                                                                                                                                                      >  1:622861‑M2/213‑301 (MQ=255)`  
                                                                                      `tgccgataatgcagttccgcactactacgctgacatctttagcgtaatggcacgtgagggcattgaccttgagttcgaagactctggtctgatgcctgacaccaaggacgtaatccgcatcctgcaagcgcgtatctatgagcaattaacgattgacctctgggaagacgcagaagacttgctcaatgaatacttggaggaagtcgaggaAGTCGCACGACAGAAAGAAATTGACCGCGCTAAGGCCCGTAAAGAACGTCACGAGGGGCGCTTAGAGGCACGCAGATTCAAACGTCGCAAc                                                                                                                                                                                                                    <  1:103716‑M2/91‑1 (MQ=255)`  
                                                                                      `tcacatggctgccgataatgcagttccgcactactacgctgacatctttagcgtaatggcaagtgagggcattgaccttgagttcgaagactctggtctgatgcctgacaccaaggacgtaatccgcatcctgcaagcgcgtatctatgagcaattaacgattgacctctgggaagacgcagaagacttgctcaatgaatacttggaggaAGTCGCACGACAGAAAGAAATTGACCGCGCTAAGGCCCGTAAAGAACGTCACGAGGGGCGCTTAGAGGCACGCAGATTCAAACGTCGCAAc                                                                                                                                                                                                                    >  1:465840‑M2/211‑301 (MQ=255)`  
                                                                                              `gacaggttccgcactactacgctgacatctttagcgtaatggcaagtgagggcattgaccttgagttcgaagactctggtctgatgcctgacaccaaggacgtaatccgcatcctgcaagcgcgtatctatgagcaattaacgattgacctctgggaagacgcagaagacttgctcaatgaatacttggaggaagtcgaggaAGTCGCACGACAGAAAGAAATTGACCGCGCTAAGGCCCGTAAAGAACGTCACGAGGGGCGCTTAGAGGCACGCAGATTCAAACGTCGCAACCGCAAGGc                                                                                                                                                                                                            <  1:517337‑M2/99‑1 (MQ=255)`  
                                                                                              `atgcagttccgcactactacgctgacatctttagcgtaatggcaagtgagggcattgaccttgagttcgaagactctggtctgatgcctgacaccaaggacgtaatccgcatcctgcaagcgcgtatctatgagcaattaacgattgacctctgggaagacgcagaagacttgctcaatgaatacttggaggaagtcgaggaAGTCGCACGACAGAAAGAAATTGACCGCGCTAAGGCCCGTAAAGAACGTCACGAGGGGCGCTTAGAGGCACGCAGATTCAAACGTCGCAACCGCAAGGc                                                                                                                                                                                                            >  1:187540‑M2/203‑301 (MQ=255)`  
                                                                                                   `gttccgcactactacgctgacatctttagcgtaattgcaagtgagggcattgaccttgagttcgaagactctggtctgatgcctgacaccaaggacgtaatccgcatcctgcaagcgcgtatctatgagcaattaacgattgacctctgggaagacgcagaagacttgctcaatgaatacttggaggaagtcgaggaAGTCGCACGACAGAAAGAAATTGACCGCGCTAAGGCCCGTAAAGAACgtcacgaggggcgcttagaggcacacagactgtctcttatacacatctccgagcccc                                                                                                                                                                                                       >  1:179109‑M2/198‑273 (MQ=255)`  
                                                                                                   `gttccgcactactacgctgacatctttagcgtaatggcaagtgagggcattgaccttgagttcgaagactctggtctgatgcctgacaccaaggacgtaatccgcatcctgctagcgcgtatctatgagcaattaacgattgacctctgggaagacgcagaagacttgctcaatgaatacttggaggaagtcgaggaAGTCGCACGACAGAAAGAAATTGACCGCGCTAAGGCACGTAAAGAACGTCACGAGGGGCGCTTAGAGGCACGCAGATTCAAACGTCGCAACCGCAAGGCACGTa                                                                                                                                                                                                       >  1:651921‑M2/198‑301 (MQ=255)`  
                                                                                                    `atgggtataagagacaggacatctttagcgtaatggcaagtgagggcattgaccttgagttcgaagactctggtatgatgcctgacaccaaggacgtaatccgcatcctgcaagcgcgtatctatgagcaattaacgattgacctctgggaagacgcagaagacttgctcaatgaatacttggaggaagtcgaggaAGTCGCACGACAGAAAGAAATTGACCGCGCTAAGGCCCGTAAAGAACGTCACGAGGGGCGCTTAGAGGCACGCAGATTCAAACGTCGCAACCGCAAGGCACGTaa                                                                                                                                                                                                      <  1:820058‑M2/105‑1 (MQ=255)`  
                                                                                                    `acaggaactactacgctgacatctttagcgtaatggcaagtgagggcattgaccttgagttcgaatactctggtctgatgcctgacaccaaggacgtaatccgcatcctgcaagcgcgtatctatgagcaattaacgattgacctctgggaagacgcagaagacttgctcaatgaatacttggaggaagtcgaggaAGTCGCACGACAGAAAGAAATTGACCGCGCTAAGGCCCGTAAAGAACGTCACGAGGGGCGCTTAGAGGCACGCAGATTCAAACGTCGCAACCGCAAGGCACGTaa                                                                                                                                                                                                      <  1:131305‑M2/105‑1 (MQ=255)`  
                                                                                                           `ctactatgctgacatctttagcgtaatggcaagtgagggcattgaccttgagttcgaagactctggtctgatgcctgacaccaaggacgtaatccgcatcctgcaagcgcgtatctatgagaaattaacgattgacctctgggaagacgcagaagacttgctcaatgaatacttggaggaagtcgaggaAGTCGCACGACAGAAAGAAATTGACCGCGCTAAGGCACGTAAAGAACGTCACGAGGGGCGCTTAGAGGCACGCAGATTCAAACGTCGCAACCGCAAGGCACGTAAAGcacac                                                                                                                                                                                               >  1:459336‑M2/190‑301 (MQ=255)`  
                                                                                                            `gttccgcactactacgctgacatctttagcgtaatggcaagtgagggcattgaccttgagttcgaagactctggtctgatgcctgacaccaaggacgtaatccgcatcctgcaagcgcgtatctatgagcaattaacgattgacctctgggaagacgcagaagacttgctcaatgaatacttggaggaAGTCGCACGACAGAAAGAAATTGACCGCGCTAAGGCCCGTAAAGAACGTCACGAGTGGCGCTTAGAGGCACGCAGATTCAAACGTCGCAACCGCAAGGCACGTAAAGcacaca                                                                                                                                                                                              >  1:195744‑M2/189‑301 (MQ=255)`  
                                                                                                             `actacgctgacatctttagcgtaatggcaagtgagggcattgaccttgagttcgaagactctggtctgatgcctgacaccaaggacgtaatccgcatcctgcaagcgcgtatctatgagcaattaacgattgacctctgggaagacgcagaagacttgctcaatgaatacttggaggaagtcgaggaAGTCGCACGACAGAAAGAAATTGACCGCGCTAAGGCCCGTAAAGAACGTCATGAGGGGCGCttagaggcacgcagattcaaacgtcgctgtctcttatacacatctccgagccc                                                                                                                                                                                             >  1:127426‑M2/188‑275 (MQ=255)`  
                                                                                                                       `catctttagcgtaatggcaagtgagggcattgaccttgagttcgaagactctggtctgatgcctgacaccaaggacgtaatccgcatcctgcaagcgcgtatctatgagcaattaacgattgacctctgggaatacgcagaagacttgctcaatgaatacttggaggaagtcgaggaAGTCGCACGACAGAAAGAAATTGACCGCGCTAAGGCCCGTAAAGAACGTCACGAGGGGCGCTTATAGGCACGCAGATTCAAACGTCGCAACCGCAAGGCACGTAAAGCACACAAAGCTAAgcgc                                                                                                                                                                                   >  1:769808‑M2/178‑301 (MQ=255)`  
                                                                                                                         `ctgacatctttagcgtaatggcaagtgagggcattgaccttgagttcgaagactctggtctgatgcctgacaccaaggacgtaatccgcatcctgcaagcgcgtatctatgagcaattaacgattgacctctgggaatacgcagaagacttgctcaatgaatacttggaggaagt‑‑T‑GCACGACAGAAAGAAATTGACCGCGCTAAGGCCCGTAAATAACGTCACGAGGGGCGCTTAGAGGCACGCAGATTCAAACGTCGCAACCGCAAGGCACGTAAAGCACACAAAGCTAAGCGCGAAAg                                                                                                                                                                              >  1:280144‑M2/176‑301 (MQ=255)`  
                                                                                                                          `ctttatcgtaatggcaagtgagggcattgaccttgagttcgaagactctggtctgatgcctgacaccaaggacgtaatccgcatcctgcaagcgcgtatctatgagcaattaacgattgacctctgggaagacgcagaagacttgctcaatgaatacttggaggaagtcgaggaAGTCGCACGACAGAAAGAAATTGACCGCGCTAAGGCCCGTAAAGAACGTCACGAGGGGCGCTTAGAGGCACGCAGATTCAAACGTCGCAACCGCAAGGCACGTAAAgcacacaaagcctgtctctta                                                                                                                                                                                >  1:204290‑M2/175‑291 (MQ=255)`  
                                                                                                                          `ctttagcgtaatggcaagtgagggcattgaccttgagttcgaagactctggtctgatgcctgacaccaaggacgtaatccgcatcctgcaagcgcgtatctatgagcaattaacgattgacctctgggaagacgcagaagacttgctcaatgaatacttggaggaagtcgaggaAGTCGCACGACAGCAAGAAATTGACCGCGCTAAGGCCCGTAAAGAACGTCACGAGGGGCGCTTAGAGGCACGCAGATTCAAACGTCGCAACCGCAAGGCACGTAAAGCACACAAAGCTAAGCGCGaa                                                                                                                                                                                >  1:136778‑M2/175‑301 (MQ=255)`  
                                                                                                                                  `taatggcaattgatggcattgaccttgagttcgaatactctggtctgatgcctgacaccaaggacgtaatccgcatcctgcaagctcgtatctatgagcaattaacgattgacctctgtgaagacgcagaagacttgctcaatgaatacttggaggaagtcgaggaAGTCGCACGACAGAAAGAAATTGACCGCGCTAAGGCCCGTAAAGAACGTCACGAGGGGCGCTTAGAGGCACGCAGATTCAAACGTCGCAACCGCAAGGCACGTAAAGCACACAAAGCTAAGCGCGAAAGAAtgct                                                                                                                                                                        <  1:716711‑M2/135‑1 (MQ=255)`  
                                                                                                                                     `tggcaagtgagggcattgaccttgagttcgaagactctggtctgatgcctgacaccaaggacgtaatccgcatcctgcaagcgcgtatctatgagcaattaacgattgacctctgggaagacgcagaagacttgctcaatgaatacttggaggaagtcgaggaAGTCGCACGACAGAAAGAAATTGACCACGCTAAGGCCCGTAAAGAACGTCACGAGGGGCGCTTAGAGGCACGCAGATtcaaacgtcgcaaccgcaaggcacgtaaagctgtctcttatccacatctccgagcccacga                                                                                                                                                                     >  1:341987‑M2/164‑271 (MQ=255)`  
                                                                                                                                     `aggcaagtgagggcatttgcctagagtacgaagactctggtctgatgcctgacaccaaggacgtaatccgcatcctgcaagcgcgtatctatgagcaattaacgattgacctctgggaagacgcagaagacttgctcaatgaatacttggaggaagtagaggaAGTCGCACGACAGAAAGAAATTGACCGCGCTAAGGCCCGTAAAGAACGTCACGAGGGGCGCTTAGAGGCACGCAGATTCAAACGTCGCAACCGCAAGGCACGTAAAGCACACAAAGCTAAGCGCGAAAGAAtgcttgc                                                                                                                                                                     <  1:272902‑M2/138‑1 (MQ=255)`  
                                                                                                                                          `ttaatggcaagtgagggcattgaccttgagttcgaagactctggtctgatgcctgacaccaaggacgtaatccgcatcctgcaagcgcgtatctatgagcaattaacgattgacctctgggaagacgcagaagacttgctcaatgaatacttggaggaAGTCGCACGACAGAAAGAAATTGACCGCGCTAAGGCCCGTAAAGAACGTCACGAGGGGCGCTTAGAGGCACGCAGATTCAAACGTCGCAACCGCAAGGCACGTAAAGCACACAAAGCTAAGCGCGAAAGAATGCTTGCTGCGt                                                                                                                                                                <  1:504928‑M2/143‑1 (MQ=255)`  
                                                                                                                                              `agggcattgacctttagttcgaagactctggtctgatgcctgacaccaaggacgtaatccgcatcctgcaagcgcgtatctatgagcaattaacgattgacctcttggaagacgcagaagacttgctcaatgaatacttggaggaagtcgaggaAGTCGCACGACAGAAAGAAATTGACCGCGCTAAGGCCCGTAAAGAACGTCACGAGGGGCGCTTAGAGGCACGCAGATTCAAACGTCGCAACCGCAAGGCACGTAAAGCACACAAAGCTAAGCGCGAAAGAATGCTTGCTGCGTGGCg                                                                                                                                                            >  1:639157‑M2/155‑301 (MQ=255)`  
                                                                                                                                                    `ataagagacaggaccttgagttcgaagactctggtctgatgcctgacaccaaggacgtaatccgcatcatgcaagcgcgtatctatgagcaattaacgattgacctctgggaagacgcagaagacttgctcaatgaatacttggaggaAGTCGCACGACAGAAAGAAATTGACCGCGCTAAGGCCCGTAAAGAACGTCACAAGGGGCGCTTAGAGGCACGCAGATTCAAACGTCGCAACCGCAAGGCACGTAAAGCACACAAAGCTAAGCGCGAAAGAATGCTTGCTGCGTGGCGATGGGc                                                                                                                                                      <  1:288765‑M2/153‑1 (MQ=255)`  
                                                                                                                                                      `aaaagtgagttcgaagactctggtctgatgcctgacaccaaggacgtaatccgcatcctgcaagcgcgtatataagagcaattaacgattgacctctgggaagacgcagaagaattgctcaatgaatacttggaggaagtggaggaAGTCGCAAGAAAGAAAGAAATAGAACGGGCTAAGGCACGAAAAGAACGTAACGAGGGGAGCTTAGAGGAACGCAGATTCAAACGTCGCAACCGCAAGGCACGTAAAGCACACAAAGCTAAGCGCGAAAGAATGCTTGCTGCGTGGCGATGGGCTg                                                                                                                                                    <  1:380539‑M2/155‑1 (MQ=255)`  
                                                                                                                                                        `gggcattgaccttgagttcgaagactctggtctgatgcctgacaccaaggacgtaatccgcatcctgcaagcgcgtatctatgagcaattaacgattgacctctgggaagacgcagaagacttgctcaatgaatacttggaggaAGTCGCACGACAGAAAGAAATTGACCGCGCTAAGGCCCGTAAAGAACGTCACGAGGGGCGCTTAGAGGCACGCAGATTCAAACGTCGCAACCGCAAGGCACGTAAAGCACACAAAGCTAAGCGCGAAAGAatgcttgctgcctgtctcttataccca                                                                                                                                                  >  1:259262‑M2/145‑288 (MQ=255)`  
                                                                                                                                                                     `gactctggtctgatgcctgacaccaaggacgtaatccgcatcctgcaagcgagtatctatgagcaattaacgattgacctctgggaagacgcagaagacttgctcaatgaatacttggaggaagtcgaggaAGTCGCACGACAGAAAGAAATTGACCGCGCTAAGGCCCGTAAAGAACGTCACGAGGGGCGCTTAGAGGCACGCAGATTCAAACGTCGCAACCGCAAGGCACGTACAGCACACAAAGCTAAGCGCGAAAGAATGCTTGCTGCGTGGCGATGGGCTGAACGTCAAGAACGGc                                                                                                                                     >  1:755775‑M2/132‑301 (MQ=255)`  
                                                                                                                                                                      `agttcgaagaatctggtctgatgccggacaccaaggacgtaatccgcatcctgcaagcgcgtatctatgagcaattaacgattgacctctgggaagacgcagaagacttgctcaatgaatacttggaggaAGTCGCACGACAGAAAGAAATTGACCGCGCTAAGGCCCGTAAAGAACGTCACGAGGGGCGCTTAGAGGCACGCAGATTCAAACGTCGCAACCGCAAGGCACGTAAAGCACACAAAGCTAAGCGCGAAAGAATGCTTGCTGCGTGGCGATGGGCTGAACGTCAAGAACGGCg                                                                                                                                    <  1:349813‑M2/171‑1 (MQ=255)`  
                                                                                                                                                                          `cggactgaggtctgacaccaaggacgtaatccgcatcctgcaagcgcgtatctatgagcaattaacgtttgacctctgggaagacgcagaagacttgctcaatgaatacttggaggaagtcgaggaAGTCGCACGACAGAAAGAAATTGACCGCGCTAAGGCCCGTAAAGAACGTCACGAGGGGAGCTTAGAGGCACGCAGATTCAAACGTCGCAACCGCAAGGCACGTAAAGCACACAAAGCTAAGCGCGAAAGAATGCTTGCTGCGTGGCGATGGGCTGAACGTCAAGAACGGCGGAAc                                                                                                                                <  1:601237‑M2/175‑1 (MQ=255)`  
                                                                                                                                                                           `gtataagagaaagcaaccaaggaggtaacccgcaaccggcaagcgcgtagctatgagcaattaacgattgacctcagggaagacgcagaagacgtgctcaatgaatacttgggggaagtcgaggaAGGCGCACGACAGAAAGAAATTGACCGCGCTAAGGCCCGAAAAGAACGTCACGGGGGGCGCTTAGAGGCACGCAGATTCAAACGTCGCAACCGCAAGGCACGTAAAGCACACAAAGCTAAGCGGGAAAAAATGCTTGCTGCGTGGCGATGGGCTGAACGTCAAGAACGGCATAAcc                                                                                                                               <  1:774181‑M2/176‑1 (MQ=255)`  
                                                                                                                                                                           `ggtctgatgcctgacaccaatgacgtaatccgcatcctgcaagcgcgtatctatgagcaattaacgattgacctctgggaagacgcagaagacttgctcaatgaatacttggatgaagtcgaggaAGTCGCACGACAGAAAGAAATTGACCGCGCTAAGGCCCGTAAAGAACGTCACGAGGGGCGCTTAGAGGCACGCAGATTCAAACGTCGCAACCGCAAGGCACGTAAAGCATACAAAGCTAAGCGCGAAAGAATGCTTGCTGCGTGGCGATGGGCTGAACGTCAAGAACGGCGTAAcc                                                                                                                               >  1:141847‑M2/126‑301 (MQ=255)`  
                                                                                                                                                                           `ggtctgatgcctgacaccaaggacgtaatccgcatcctgcaagcgcgtatctatgagcaattaacgattgacctctgggaagacgcagaagacttgctcaatgaatacttggaggaagtcgaggaAGTCGCACGACAGAAAGAAATTGACCGCGCTAAGGCCCGTAAAGAACGTCACGAGGGGCGCTTAGAGGCACGAAGATTCAAAAGTCGCAACCGCAAGGCACGTAAAGCACACAAAGCTAAGCGCGAAAGAATGCTTGCTGCGTGGCGATGGGCTGAACGTCAAGAACGGCGTAAcc                                                                                                                               >  1:529142‑M2/126‑301 (MQ=255)`  
                                                                                                                                                                           `ggtctgatgcctgacaccaaggacgtaatccgcatcctgcaagcgcgtatctatgagcaatgaacgattgacctcggggaagacgcagaagacttgctcaatgaatacttggaggaagtcgaggaAGTCGCACGACAGAAAGAAATTGACCGCTCTAAGGCCCGTAAAGAACGTCACGAGGGGCGCTTAGAGGCACGCAGATTCAAATGTAGCAACCGCAAGGCACGTAAAGCACACAAAGCTAAGCGCGAAAGAATGCTTGCTGCGTGGCGATGGGCTGAACGTCAAGAACGGCgtaaac                                                                                                                               >  1:709715‑M2/126‑299 (MQ=255)`  
                                                                                                                                                                            `gtctgatgcctgacaccaagtacgtaatccgcatcctgcaagcgcgtagctatgagcaattaacgattgacctctgggaagacgcagaagacttgctcaatgaatacttggaggaagtcgaggaAGTCGCACGACAGAAAGAAATTGACCGCGCTAAGGCCCGTAAAGAACGTCACGAGGGGCGCTTAGAGGCACGCAGATTCAAACGTCGCAACCGCAAGGCACGTAAAGCACACAAAGCTAAGCGCGAAAGAATGCTTGCTGCGTGGCGATGGGCTGAACGTCAAGAACGGCGTAACCa                                                                                                                              <  1:823241‑M2/177‑1 (MQ=255)`  
                                                                                                                                                                                  `tgcctgacaccaaggacgtaatccgcatcctgcaagcgcgtatctatgagcaattaacgattgacctctgggaagacgcagaagacttgctcaatgaatacttggaggaagtcgaggaAGTCGCACGACAGAAAGAAATTGACCGCGCTAAGGCCCGTAAAGAACGTCACGAGGGGCGCTTAGAGGCACGCAGATTCAAACGTCGCAACCGCAAGGCACGTAAAGCACACAAAGCTAAGCGCGAAAGAATGCTTGCTGCGTGGCGATGGGCTGAACGTCAAGAACGGCGTAACCATGAGGt                                                                                                                        <  1:789983‑M2/183‑1 (MQ=255)`  
                                                                                                                                                                                       `gacaccaaggacgtaagccgcatccggcaagcgcgtatctatgagcaattaacgattgacctcttggaagacgcagaagacttgctcaatgaatacttggaggaagtcgaggaAGTCGCACGACAGAAAGAAATTGACCGCGCTAAGGCCCGTAAAGAACGTCACGAGGGGCGCTTAGAGGCACGCAGATTCAAACGTCGCAACCGCAAGGCACGTAAAGCACACAAAGCTAAGCGCGAAATAATGCTTGCTGCGTGGCGATGGGCTGAACGTCAAGAACGGCGTAACCATGAGGTAGCTg                                                                                                                   <  1:164218‑M2/188‑1 (MQ=255)`  
                                                                                                                                                                                         `caccaaggacgtaatccgcatcctgcaagcgcgtatctatgagcaattaacgattgacctctgggaagacgcagaagacttgctcaatgaatacttggaggaagtcgaggaAGTCGCACGACAGAAAGCAATTGACCGCGCTAAGGCCCGTAAAGAACGTCATTAGGGGCGCTTCGAGGCACGCAGATTCAAACGTCGCAACCGCAAGGCACGTAAAGCACACACAGCTAAGCGCGAAAGAATGCTTGCTGCGTGGCGATGGGCTGAACGTCAAGAACGGCGTAACCATGAGGTAGCTgtt                                                                                                                 >  1:801164‑M2/112‑300 (MQ=255)`  
                                                                                                                                                                                           `ccacggacgtaatccgcatcctgcaagcgcgtatctatgagcaattaacgattgacctctgggaagacgcagaagacttgctcaatgaatacttggaggaagtcgaggaAGTCGCACGACAGAAAGAAATTGACCGCGCTAAGGCCCGTAAAGAACGTCACGAGGGGCGCTTAGAGGCACGCAGATTCAAACGTAGCAACCGCAAGGCACGTAAAGCACACAAAGCTAAGCGCGAAAGAATGCTTGCTGCGTGGCGATGGGCTGAACGTCAAGAACGGCGTAACCATGAGGTAGCTGTAGa                                                                                                               >  1:268837‑M2/110‑301 (MQ=255)`  
                                                                                                                                                                                            `agatgtgtataagagacagctgcaagtgcgtatctatgagcaattaactattgacctctgggaagacgcagaagacttgctcaatgaatacttggaggaagtcgaggaAGTCGCACGACAGAAAGAAATTGACCGCGCTAAGGCCCGTAAAGAACGTCACGAGGGGCGCTTAGAGGCACGCAGATTCAAACGTCGCAACCGCAAGGCACGTAAAGCACACAAAGCTAAGCGCGAAAGAATGCTTGCTGCGTGGCGATGGGCTGAACGTCAAGAACGGCGTAACCATGAGGTAGCTGTAGAt                                                                                                              <  1:320730‑M2/193‑1 (MQ=255)`  
                                                                                                                                                                                             `aaggacggaatctgcatcctgcaagcgcgtatctatgagcaattaacgattgacctctgggaagactcagaagacttgctcaatgaatacttggaggaagtcgaggaAGTCGAACGACAGAAAGAAATTGACCGCGCTAAGGCCCGTAAAGAACGTCACGAGGGGCGCTTAGAGGCACGCAGATTCAAACGTCGCAACCGCAAGGCACGTAAAGCACACAAAGCTAAGCGCGAAAGAATGCTTGCTGCGTGGCGATGGGCTGAACGTCAAGAACGGCGTAACCATGAGGTAGCTGTAGATg                                                                                                             <  1:181721‑M2/194‑1 (MQ=255)`  
                                                                                                                                                                                                `gacgtaatccgcatcctgcaagcgcgtatctatgagcaattaacgattgacatctgggaagacgcagaagacttgctcaatgaatacttggaggaagtcgaggaAGTCGCACGACAGAAAGAAATTGACCGCGCTAAGGCCCGTAAAGAACGTCACGAGGGGCGCTTAGAGGCACGCAGATTCAAACGTCGCAACCGCAAGGCACGTAAAGCACACAAAGCTAAGCGCGAAAGAATGCTTGCTGCGTGGCGATGGGCTGAACGTCAAGAACGGCGTAACCATGAGGTAGCTGTAGATGTAc                                                                                                          <  1:574928‑M2/197‑1 (MQ=255)`  
                                                                                                                                                                                                `aacaccaaggacgtaatccgcaacctgcaagggcggatctaagagcaattaacgattgaactcttggaagacgaagaagaattgctaaatgaatacttggaggaAGTCGCACGAAAGAAAGAAATTGACCGGGCTAAGGCCCGTAAAGAACGTCACGAGGGGCGCTTAGAGGCACGCAGATTCAAACGTCGCAACCGCAAGGCACGTAAAGCACACAAAGCTAAGCGCGAAAGAATGCTTGCTGCGTGGCGATGGGCTGAACGTCAAGAACGGCGTAACCATGAGGTAGCTGTAGATGTAc                                                                                                          <  1:610668‑M2/197‑1 (MQ=255)`  
                                                                                                                                                                                                   `gtaatccgcgtcctgcaagcgcgtatctatgagcaattaacgattgacctctgggaagacgcagaagacttgctcaatgaatacttggaggaagtcgaggaAGTCGCACGACAGAAAGAAATTGACCGCGCTAAGGCCCGTAAAGAACGTCACGAGGGGCGCTTAGAGGCACGCAGATTCAAACGTCGCAACCGCAAGGCACGTAAAGCACACAAAGCTAAGCGCGAAAGAATGCTTGCTGCGTGGCGATGGGCTGAACGTCCAGAACGGCGTAACCATGAGGTAGCTGTAGATGTACTAg                                                                                                       >  1:373041‑M2/102‑301 (MQ=255)`  
                                                                                                                                                                                                     `caaggacgtaatccgcatcctgcaagcgcgtatcgatgagcaattaacgattgacctctggtaagacgtagaagacttgctcaatgaatacttggaggaAGTCGCACGACAGAAAGAAATTGACCGCGCTAAGGCCCGTAAAGAACGTCACGAGGGGCGCTTAGAGGCACGCAGATTCAAACGTCGCAACCGCAAGGCACGTAAAGCACACAAAGCTAAGCGCGAAAGAATGCTTGCTGCGTGGCGATGGGCTGAACGTCAAGAACGGCGTAACTATGAGGTAGCTGTAGATGTACTAGga                                                                                                     <  1:63150‑M2/202‑1 (MQ=255)`  
                                                                                                                                                                                                     `aggccgcatcctgcaagcgcgtatctatgagcaattaacgattgacctctgggaagacgcagaagacttgctcaatgaatacttggaggaagtcgaggaAGTCGCACGACAGAAAGAAATTGACCGCGCTAAGGCCCGTAAAGAACGTCACGAGGGGCGCTTAGAGGCACGCAGATTCAAACGTCGCAACCGCAAGGCACGTAAAGCACACAAAGCTAAGCGCGAAAGAATGCTTGCTGCGTGGCGATGGGCTGAACGTCAAGAACGGCGTAACCATGAGGTAGCTGTAGATGTACTAGga                                                                                                     <  1:216858‑M2/202‑1 (MQ=255)`  
                                                                                                                                                                                                     `aatccgcatcctgcaagcgcgtatctatgagcaattaacgattgacctctgtgaagacgcagaagacttgctcaatgaatacttggaggaagtcgaggaAGTCGCACGACAGAAAGAAATTGACCGCGCTAAGGCCCGTAAAGAACGTCACTATGGGCGCTTAGAGGCACGCAGATTCAAACGTCGCAACCGCAAGGCACGTAAAGCACACAAAGCTAAGCGCGAAAGAATGCTTGCTGCGTGGCGATGGGCTGAACGTCAAGAACGGCGTAACCATGAGGTAGCTGTAGATGTACTAGga                                                                                                     <  1:337302‑M2/202‑1 (MQ=255)`  
                                                                                                                                                                                                     `aatccgcatcctgcaagcgcgtatctatgagcaattaacgattgacctctgggaagacgcagaagacttgctcaatgaatacttggaggaagtcgaggaAGTCGCACGACAGAAAGAAATTGACTGCGCTAAGGCCCGTAAAGAACGTCACGAGGGGCGCTTAGAGGCACGCAGATTCAAACGTCGCAACCGCAAGGCACGTAAAGCACACAAAGCTAAGCGAGAAAGAATGCTTGCTGCGTGGCGATGGGCTGAACGTCAAGAACGGCGTAACCATGAGGTAGCTGTAGATGTACTAGga                                                                                                     <  1:436023‑M2/202‑1 (MQ=255)`  
                                                                                                                                                                                                     `aatccgcatcctgcaagcgcgtatctatgagcaattaacgattgacctctgggaagacgcagaagacttgctcaatgaatacttggaggaagtcgaggaAGTCGCACGACAGAAAGAAATTGACCTCGCTAAGGCCCGTAAAGAACGTCACGAGGGGCGCTTAGAGGCACGCAGATTCAAACGTCGCAACCGCAAGGCACGTAAAGCACACAAAGCTAAGCGCGAAAGAATGCTTGCTGCGTGGCGATGGGCTGAACGTCAAGAACGGCGTAACCATGAGGTAGCTGTAGATGTACTAGga                                                                                                     <  1:591175‑M2/202‑1 (MQ=255)`  
                                                                                                                                                                                                       `tccgcatcctgcaagcgcgtatctatgagcaattaacgattgacctcttggacgacgcagaagacttgctcaatgaatacttggaggaagtcgaggaAGTCGCACGACAGAAAGAAATTGACCGCGCTAAGGCCCGTAAAGAACGTCACGAGGGGCGCTTAGCGGCACGCAGATTCAAACGTCGCAACCGCAAGGCACGTACAGCACACAACGCTAAGCGCGAAAGAATGCTTGCTGCGTGGCGATGGGCTGAACGTCAAGAACGGAGTAATCATGAGGTAGCTGTAGAGGTACTAGgaag                                                                                                   >  1:596330‑M2/98‑301 (MQ=255)`  
                                                                                                                                                                                                       `tccgcatcctgcaagcgcgtatctatgagcaattaacgattgacctctgggaagacgcagaagacttgctcaatgaatacttggaggaagtcgaggaAGTCGCACGACAGAAAGAAATTGACCGCGCTAAGGCCCGTAACGAACGTCACGAGGGGCGCTTAGAGGCACGCAGATTCAAACGTCGCAACCGCAAGGCACGTAAAGCACACAAAGCTAAGCGCGAAAGAATGCTTGCTGCGTGGCGATGGGCTGAACGTCAAGAACGGCGTCACCCTGAGGTAGCTGTAGATGTACTAGgaag                                                                                                   >  1:370762‑M2/98‑301 (MQ=255)`  
                                                                                                                                                                                                                        `cgtatctatgagcaattaacgattgaccgctgggaagacgcagaagacttgctcaatgaatacttggaggaaatcgaggaAGTCGCACGACAGAAAGAAATTGACCGCGCTAAGGACCGTAAAGAACGTCACGAGGGGCGCTTAGAGGCACGCAGATTCAAACGTCGCAACCGCAAGGCACGTAAAGCACACAAAGCTAAGCGCGAAAGAATGCTTGCTGCGTGGCGATGGGCTGAACGTCAAGAACGGCGTAACCATGAGGTAGCTGTAGATGTACTAGGAAGAACCAATAACGCTATGc                                                                                  <  1:211402‑M2/221‑1 (MQ=255)`  
                                                                                                                                                                                                                          `tatctatgaggaattaacgattgacctctgggaagacgcagaagacttgctcaatgaatacttggaggaagtctaggaAGTCGCACGACAGAAAGAAATTGACCGCGCTAAGGCCCGTAAAGAACGTCACGAGTGGCGCTTAGAGGCACGCAGATTCAAACGTCGCAACCGCAAGGCACGTAAAGCACACAAAGCTAAGCGCGAAAGAATGCTTGCTGCGTGGCGATGGGCTGAACGTCAAGAACGGCGTAACCATGAGGTAGCTGTAGATGTACTAGGAAGAACCAATAACACTATGctc                                                                                <  1:172719‑M2/223‑1 (MQ=255)`  
                                                                                                                                                                                                                          `tatctatgagcaattaacgattgacctgtgggaagacgcagaagacttgctcaatgaatacttggaggaagtcgaggaAGTCGCACGACAGAAAGAAATTGACCGCGCTAAGGCCCGTAAAGAACGTCACGAGGGGCGCTTAGAGGCACGCAGATTCAAACGTCGCAACCGCAAGGCACGTAAAGCACACAAAGCTAAGCGCGAAAGAATGCTTGCTGCGTGGCGATGGGCTGAACGTCAAGAACGGCGTAACCATGAGGTAGCTGTAGATGTACTAGGAAGAACCAATAACGCTATGctc                                                                                <  1:733024‑M2/223‑1 (MQ=255)`  
                                                                                                                                                                                                                          `tatctatgagcaattaacgattgacctctgggaagacgcagaagacttgctcaatgaatacttggaggaagtcgaggaAGTCGCACGACAGAAAGAAATTGACCGGGCTAAGGCCCGTAAAGAACGTCACGAGGGGCGCTTAGAGGCACGCAGATTCAAACGTCGCAACCGCAAGGCACGTAAAGCACACAAAGCTAAGCGCGAAAGAATGCTTGCTGCGTGGCGATGGGCTGAACGTCAAGAACGGCGTAACCATGAAGTAGCTGTAGATGTACTAGGAAGAACCAATAACGATATGctc                                                                                <  1:119167‑M2/223‑1 (MQ=255)`  
                                                                                                                                                                                                                            `tctattagcaattaacgattgacctctgggaagacgcagaagacttgctcaatgaatacttggaggaagtcgaggaAGTCGCACGACAGAAAGAAATTGACCGCGCTAAGGCCCGTAAAGAACGTCACGAGGGGCGCTTAGATGCACGCAGATTCAAACGTCGCAACCGCAAGGCACGTAAAGCACACAAAGCTAAGCGCGAAAGAATGCTTGCTGCGTGGCGATGGGCTGAACGTCAAGAACGGCGTAACCATGAGGTAGCTGTAGATGTACTAGGAAGAACCAATAACGCTATGCTCTg                                                                              >  1:248291‑M2/77‑301 (MQ=255)`  
                                                                                                                                                                                                                            `tctatgagcaattaacgattgacctctgggaagacgcagaagacttgctcaatgaatacttggaggaagtcgaggaAGTCGCACGACAGAAAGAAATTGACCGCGCTAAGGCCCGTAAAGAACGTCACGAGGGGCGCTTAGAGGCACGCAGATTCAAACGTCGCAACCGCAAGGCACGTAAAGCACACAAAGCTAAGCGCGAAAGAATGCTTGCTGCGTGGCGATGGGCTGAACGTCAAGAACGGCGTAACCATTAGGTAGCTGTAGATGTACTAGGAAGAACCCATAACGCTATGCTCTg                                                                              >  1:82643‑M2/77‑301 (MQ=255)`  
                                                                                                                                                                                                                                   `tatctatgagcaattaacgattgacttctgggaagacgcagaagacttgctcaatgaatacttggaggaAGTCGCACGACAGAAAGAAATTGACCGCGCTAAGGCCCGTAAAGAACGTCACGAGGGGCGCTTAGAGGCACGCAGATTCAAACGTCGCAACCGCAAGGCACGTAAAGCACACAAAGCTAAGCGCGAAAGAATGCTTGCTGCGTGGCGATGGGCTGAACGTCAAGAACGGCGTAACCATGAGGTAGCTGTAGATGTACTAGGAAGAACCAATAACGCTATGCTCTGGGTCAAc                                                                       <  1:303943‑M2/232‑1 (MQ=255)`  
                                                                                                                                                                                                                                   `gcacttaacgattgacatctgggaagacgcagaagacttgctcaatgaatacttggaggaagtcgaggaAGTCGCACGACAGAAAGAAATTGACCGCGCTAAGGCCCGTAAAGAACGTCACGAGGGGCGCTTAGAGGCACGCAGATTCAAACGTCGCAACCGCAAGGCACGTAAAGCACACAAAGCTAAGCGCGAAAGAATGCTTGCTGCGTGGCGATGGGCTGAACGTCAAGAACGGCGTAACCATGAGGTAGCTGTAGATGTACTAGGAAGAACCAataacactatgcctgtctcttat                                                                       >  1:396731‑M2/70‑290 (MQ=255)`  
                                                                                                                                                                                                                                   `gatgtgtataagagacagctgggaagacgcagaagacttgctcaatgaatacttggaggaagtcgaggaAGTCGCACGACAGAAAGAAATTGACCGCGCTAAGGCCCGTAAAGAACGTCACGAGGGGCGCTTAGAGGCACGCAGATTCAAACGTCGCAACCGCAAGGCACGTAAAGCACACAAAGCTAAGCGCGAAAGAATGCTTGCTGCGTGGCGATGGGCTGAACGTCAAGAACGGCGTAACCATGAGGTAGCTGTAGATGTACTAGGAAGAACCAATAACGCTATGCTCTGGGTCAAc                                                                       <  1:725454‑M2/232‑1 (MQ=255)`  
                                                                                                                                                                                                                                    `agattaatgattgacctctgggaagacgcagaagacttgctcaatgaatacttggaggaagtcgaggaAGTCGCACGACAGAAAGAAATTGACCGCGCTAAGGCCCGTAAAGAACGTCACGAGGGGCGCTTAGAGGCACACAGATTCAAACGTCGCAACCGCAAGGCACGTAAAGCACACAAAGCTAAGCGCGAAAGAATGCTTGCTGCGTGGCGATGGGCTGAACGTCAAGAACGGCGTAACCATGAGGTAGCTGTAGATGTACTAGGAAGAACCAATAACGCTATGCTCTGAGTCAACa                                                                      <  1:474517‑M2/233‑1 (MQ=255)`  
                                                                                                                                                                                                                                      `attaacgattgacctctgggaagacgcagaagacttgctcaatgaatacttggaggaagtcgaggaAGTCGCACGACAGAAAGAAATTGACCGCGCTAAGGCCCGTAAAGAACGTCACGAGGGGCGCTTAGAGGCACGCAGATTCAAACGTCGCAACCGCAAGTCACGTAAAGCACACAAAGCTAAGCGCGAAAGAATGCTTGCTGCGTGGCGATTGGCTGAACGTCAAGAACGGCGTAACCATGAGGTAGCTGTAGATGTACTAGGAAGAACCAATAACGCTATGCTCTGGGTCAACATg                                                                    >  1:798752‑M2/67‑301 (MQ=255)`  
                                                                                                                                                                                                                                         `ggggctcggagatgtgtataagagacaggacttgctcaatgaatacttggaggaagtcgaggaAGTCGCACGACAGAAAGAAATTGACCGCGCTAAGGCCCGTAAAGAACGTCACGAGGGGCGCTTAGAGGCACGCAGATTCAAACGTCGCAACCGCAAGGCACGTAAAGCACACAAAGCTAAGCGCGAAAGAATGCTTGCTGCGTGGCGATGGGCTGAATGTCAAGAACGGCGTAACCATGAGGTAGCTGTAGATGTACTAGGAAGAACCAATAACGCTATGCTCTGGGTCAACATGTtc                                                                 <  1:155373‑M2/238‑1 (MQ=255)`  
                                                                                                                                                                                                                                           `cgattgacctcttggaagacgcagaagacttgctcaatgaatacttggaggaagtcgaggaAGTCGCACGACAGAAAGAAATTGACCGCGCTAAGGCCCGTAAAGAACGTCACGAGTGGCGCTTAGAGGCACGCAGATTCAAACGTCGCAACCGCAAGGCACGTAAAGCACACAAAGCTAAGCGCGAAAGAATGCTTGCTGCGTGGCGATGGGCTGAACGTCAAGCACGGCGTAACCATGAGGTAGCTGTAGATGTactaggaagaaccaataacgctactgtctcttatacacatctccc                                                               >  1:677948‑M2/62‑279 (MQ=255)`  
                                                                                                                                                                                                                                           `agattggcctctgggaagacgcagaagacttgctcaatgaatacttggaggaagtcgaggaAGTCGCACGACAGAAAGAAATTGACCGCGCTAAGGCCCGTAAAGAACGTCACGAGGGGCGCTTAGAGGCACGCAGATTCAAACGTCGCAACCGCAAGGCACGTAAAGCACACAAAGCTAAGCGCGAAAGAATGATTGCTGCGTGGCGATGGGCTGAACGTCAAGAACGGCGTAACCATGAGGTAGCTGTAGATGTACTAGGAAGAACCAATAACGCTATGCTCTGGGTCAACATGTtctc                                                               <  1:604780‑M2/240‑1 (MQ=255)`  
                                                                                                                                                                                                                                                `tgtataagagacagcgcagaagacttgctcaatgaatacttggaggaagtcgaggaAGTCGCACGACAGAAAGAAATTGACCGCGCTAAGGCCCGTAAAGAACGTCACGAGGGGCGCTTAGAGGCACGCAGATTCAAACGTCGCAACCGCAAGGCACGTAAAGCACACAAAGCTAAGCGCGAAAGAATGCTTGCTGCGTGGCGATGGGCTGAACGTCAAGAACGGCGTAACCATGAGGTAGCTGTAGATGTACTAGGAAGAACCAATAACGCTATGCTCTGGGTCAACATGTTCTCTgggg                                                          <  1:431998‑M2/245‑1 (MQ=255)`  
                                                                                                                                                                                                                                                `gtataagagacagacgaagaagacttgctcaatgaatacttggaggaagtcgaggaAGTCGCACGACAGAAAGAAATTGACCGCGATAAGGCCCGTAAAGAACGTCACGAGGGGCGCTTAGAGGCACGCAGATTCAAACGTCGCAACCGCAAGGCACGTAAAGCACACAAAGCTAAGCGCGAAAGAATGCTTGCTGCGTGGCGATGGGCTGAACGTCAAGAACGGCGTAACCATGAGGTAGCTGTAGATGTACTAGGAAGAACCAATAACGCTATGCTCTGGGTCAACATGTTCTCTgggg                                                          <  1:760100‑M2/245‑1 (MQ=255)`  
                                                                                                                                                                                                                                                  `gtgggctcggagatgtgtataagagacagatgaatacttggaggaagtcgaggaAGTCGCACGACAGAAAGAAATTGACCGCGCTAAGGCCCGTAAAGAACGTCACGAGGGGCGCTTAGAGGCACGCAGATTCAAACGTCGCAACTGCAAGGCACGTAAAGCACACAAAGCTAATCGCGAAAGAATGCTTGCTGCGTGGCGATGGGCTGAACGTCAAGAACGGCGTAACCATGAGGTAGCTGTAAATGTACTAGGAAGAACCAATAACGCTATGCTCTGGGTCAACATGTTCTCTGGGGAc                                                        <  1:661509‑M2/247‑1 (MQ=255)`  
                                                                                                                                                                                                                                                   `ctctgtgaagacgcagaagacttgctcaatgaatacttggaggaagtcgaggaAGTCGCACGACAGAAAGAAATTGACCGCGCTAAGGCCCGTAAAGAACGTCACGAGGGGCGCTTAGAGGCACGCAGATTCAAACGTCGCAACCGCAAGGCACGTAAAGCACACAAAGCTAAGCGCGAAAGAATGCTTGCTGCGTGGCGATGGGCTGAACGTCAAGAACGGCGTAACCATGAGGTAGCTGTAGATGTACTAGGAAGAACCAATAACGCTATGCTCTGGgtcaacatgttcctgtctctta                                                       >  1:669928‑M2/54‑291 (MQ=255)`  
                                                                                                                                                                                                                                                     `gattgacctctgggaagacgcagaagacttgctcaatgaatacttggaggaAGTCGCACGACAGAAAGAAATTGACCGCGCTAAGGCCCGTAAAGAACGTCACGAGGGGCGCTTAGAGGCACGCAGATTCAAACGTCGCAACCGCAAGGCACGTATAGCACACAAAGCTAAGCGCGAAAGAATGCTTGCTGCGTGGCGATGGGCTGAACGTCAAGAACGGCGTAACCATGAGGTAGCTGTAGATGTACTAGGAAGAACCAATAACGCTATGCTCTGGGTCAACATGTTCTCTGGGGACttt                                                     <  1:233454‑M2/250‑1 (MQ=255)`  
                                                                                                                                                                                                                                                     `cttggaagacgcagaagacttgctcaatgaatacttggaggaagtcgaggaAGTCGCACGACAGAAAGAAATTGACAGCGCTAAGGCACGTAAAGAACGTCACGAGGGGCGCTTAGAGGCACGCAGATTCAAACGTCGCAACCGCAAGGCACGTAAAGCACACAAAGCTAAGCGCGAAAGAATGCTTGCTGCGTGGCGATGGGCTGAACGTCAAGAACGGCGTAACCATGAGGTAGCTGTAGATGTACTAGGAAGAACCAATAACGCTATGCTCTGGgtcaacatgttctcctgtctcttt                                                     >  1:420189‑M2/52‑291 (MQ=255)`  
                                                                                                                                                                                                                                                          `gagatgtttattagggacaaaatgaatacttggagggagtcgaggaAGTCGCACGACAGAAAGGAATTGACCGCGCTTAGGCCCGTAAAGAACGTCACGGGGGGCGCTTAGAGGCACGCAGATTCAAACGTCGCAACCGCAAGGCACGGAAAGCCCACAAAGCTAAGCGCGGAAGAATGCTTGCTGCGTGGCGATTGGCTGAACGTCCAGAACGGCGTAACCATGGGGTAGCTGTTGATGGTCTAGGAAGAACCAATAACGCTATGCTCTGGGTCAACATGTTCTCTGGGGACTTTAAGgc                                                <  1:107061‑M2/255‑1 (MQ=255)`  
                                                                                                                                                                                                                                                                `cagaagacttgctcaatgaatacttggaggaagtcgaggaAGTCGCACGACAGAAAGAAATTGACCGCGCTAAGGCCCGTAAAGAACGTCACGAGGGGCGCTTAGAGGCACGCAGATTCAAACGTCGCAACCGCAAGGCACGTAAAGCACACAAAGCTAAGCGCGAAAGAATGCTTGCTGCGTGGCGACGGGCTGAACGTCAAGAACGGCGTAACCATGAGGTAGCTGTAGATGTACTAGGAAGAACCAATAACGCTATGCTCTGGGTCAACATGTTCTCTGGGGACTTTAAGGCGCTTGa                                          >  1:361511‑M2/41‑301 (MQ=255)`  
                                                                                                                                                                                                                                                                             `caatgaatacttggaggaagtcgaggaAGTCGCACGACAGAAAGAAATTGACCGCGCTAAGGCCCGTAAAGAACGTCACGAGGGGCGCTTAGAGGCACGCAGATTCAAACGTCGCAACCGCAAGGCACGTAAAGCACCCAAAGCTAAGCGCGAAAGAATGCTTGCTGCGTGGCGATGGGCTGAACGTCAAGAACGGCGTAACCATGAGGTAGCTGTAGATGTACTAAGAAGAACCAATAACGCTATGCTCTGGGTCAACATGTTCTCTGGGAACTTTAAGGCGCTTGAGGAACGAATcgcg                             >  1:329286‑M2/28‑301 (MQ=255)`  
                                                                                                                                                                                                                                                                              `aatgaatacttggaggaagtcgaggaAGTCGCACGACAGAAAGAAATTGACCGCGCTAAGGCCCGTAAAGAACGTCACGAGGGGCGCTTAGAGGCACGCAGATTCAAACGTCGCAACCGCAAGGCACGTAAAGCACACAAAGCTAAGCGCGAAAGAATGCTTGCTGCGTGGCGATGGGCTGAACGTCAAGAACGGCGTAACCATGAGGTAGCTGTAGATGTACTAGGAAGAACCAATAACGCTATGCTCTGGGTCAACATGTTCTCTGGGGACTTTAAGGCGCTTGATGAACGAATCgcgc                            <  1:100399‑M2/275‑1 (MQ=255)`  
                                                                                                                                                                                                                                                                               `atgaatacttggaggaagtcgaggaAGTCGCACGACAGAAAGAAATTGACCGCGCTAAGGCCCGTAAAGAACGTCACGAGGGGCGCTTAGAGGCACGCAGATTCAAACGTCGCAACCGCAAGGCACGTAAAGCACACAAAGCTAAGCGCGAAAGAATGCTTGCTGCGTGGCGATGGGCTGAACGTCAAGAACGGCGTAACCATGAGGTAGCTGTAGATGTACTAGGAAGAACCAATAACGCTATGCTCTGGGTAAACATGTTCTCTGGGGACTTTAAGGAGCTTGAGGAACGAATCGCGCt                           >  1:242312‑M2/26‑301 (MQ=255)`  
                                                                                                                                                                                                                                                                               `atgaatacttggaggaagtcgaggaAGTCGCACGACAGAAAGAAATTGACCGCGCTAAGGCCCGTAAAGAACGTCACGAGGGGCGCTTAGAGGCACGCAGATTCAAACGTCGCAACCGCAAGGCACGTAAAGAACACAAAGCTAAGCGCGAAAGAATGCTTGCTGCGTGGCGATGGGCTGAACGTCAAGAACGGCGTAACCATGAGGTAGCTGTCGATGTACTAGGAAGAACCAATAACGCTATGCTCTGGGTAAACATGTTCTCTGGGGCCTTTAAGGCGCTTGAGGAACGAATCGCGCt                           >  1:390027‑M2/26‑301 (MQ=255)`  
                                                                                                                                                                                                                                                                                      `cttggaggaagtcgaggaAGTCGCACGACAGAAAGAAATTGACCGCGCTAAGGCCCGTAAAGAACGTCACGAGGGGCGCTTAGAGGCACGCAGATTCAAACGTCGCAACCGCAAGGCACGTAAAGCACACAAAGCTAAGCGCGAAAGAATGCTTGCTGCGTGGCGATGGGCTGAACGTCAAGAACGGCGTAACCATGAGGTAGCTGTAGATGTACTAGGAAGAACCAAAAACGCTATGCTCTGggtcaacatgttctctggggactttaaggcccggcccgtataaacaactccgagccca                    >  1:784985‑M2/19‑273 (MQ=255)`  
                                                                                                                                                                                                                                                                                        `tgtaggaagtcgaggaAGTCGCACGAAAGAAAGAAATTGACCGCGCTAAGGCCCGTAAAGAACGTCACGAGGGGCGCTTAGAGGCACGCAGATTCAAACGTCGCAACCGCAAGGCACGTAAAGCACACAAAGCTAAGCGCGAAAGAATGCTTGCTGCGTGGCGATGGGCTGAACGTCAAGAACGGCGTAACCATGAGGTAGCTGTAGATGTACTAGGAAGAACCAATAACGCTATGCTCTGGGTCAACATGTTCTCTGGGGACTTTAAGGCGCTTGAGGAACGAATCGCGCTGCACTGGCg                  <  1:121331‑M2/285‑1 (MQ=255)`  
                                                                                                                                                                                                                                                                                         `aagatacttggaggaAGTCGCACGACAGAAAGAAATTGACCGCGCTAAGGCCCGTAAAGAACGTCACGATGGGCGCTTAGAGGCACGCAGATTCAAACGTCGCAACCGCAAGGCACGTAAAGCACACAAAGCTAAGCGCGAAAGAATGCTTGCTGCGTTGCGATGGGCTGAACGTCAAGAACGGCGTAACCATGAGGTAGCTGTAGATGTACTAGGAAGAACCAATAACGCTATGCTCTGGGTCAACATGTTCTCTGGGGACTTTAAGGCGCTTGAGGAACGAATCGCGCTGCACTGGCGt                 <  1:484985‑M2/286‑1 (MQ=255)`  
                                                                                                                                                                                                                                                                                              `acttggaggaAGTCGCACGACAGAAAGAAATTGACCGCGCTAAGGCCCGTAAAGAACGTCACGAGGGGCGCTTAGAGGCACGCAGATTCAAACGTCGCAACCGCAAGGCACGTAAAGCACACAAAGCTAAGCGCGAAAGAATGCTTGCTGCGTGGCGATGGGCTGAACGTCAAGAACGGCGTAACCATTAGGTAGCTGTAGATGTACTAGGAAGAACCAATAACGCTATGCTCTGGGTCAACATGTTCTCTGGGGACTTTAAGGCGCTTGAGGAACGAATCGCGCTGCACTGGCGTAATGc            <  1:549119‑M2/291‑1 (MQ=255)`  
                                                                                                                                                                                                                                                                                              `aagtcgaggaAGGCGAACGACAGAAAGAAATTGACCGCGCTAAGGCCCGTAAAGAACGTCACGAGGGGCGCTTAGAGGCACGCAGATTCAAACGTCGCAACCGCAAGGCACGTAAAGCACACAAAGCTAAGCGCGAAAGAATGCTTGCTGCGTGGCGATGGGCTGAACGTCAAGAACGGCGTAACCATGAGGTAGCTGTAGATGTACTAGGAAGAACCAATAACGCTATGCTCTGGGTCAACATGTTCTCTGGGGACTTTAAGGCGCTTGAGGAACGAATCGCGCTGCACTGGCGTAATGc            <  1:410155‑M2/291‑1 (MQ=255)`  
                                                                                                                                                                                                                                                                                              `aaggcgaggaAGTCGAACGACAGAAAGAAATTGACCGCGCTAAGGCCCGTAAAGAACGTCACGAGTGGCGCTTAGAGGCACGCAGATTCAAACGTCGCAACCGCAAGGCAAGTAAAGCACACAAAGCTAAGCGCGAAAGAATGCTTGCTGCGTGGCGATGGGCTGAACGTCAAGAACGGCGTAACCATGAGGTAGCTGTAGATGTACTAGGAAGAACCAATAACGCTATGCTCTGGGTCAACATGTTCTCTGGGGACTTTAAGGCGCTTGAGGAACGAATCGCGCTGCACTGGCATAATGc            <  1:781594‑M2/291‑1 (MQ=255)`  
                                                                                                                                                                                                                                                                                                        `agtcgTACGACAGAAAGAAATTGACCGCGCTAAGGCCCGTAAAGAACGTCACGAGGGGCGCTTAGAGGCACGCAGATTCAAACGTCGCAACCGCAAGGCACGTAAAGCACACAAAGCTAAGCGCGAAAGAATGCTTGCTGCGTGGCGATGGGCTGAACGTCAAGAACGGCGTAACCATGAGGTAGCTGTAGATGTACTAGGAAGAACCAATAACGCTATGCTCTGGGTCAACATGTTCTCTGGGGACTTTAAGGCGCTTGAGGAACGAATCGCGCTGCACTGGCGTAATGCTGACCAGATg  <  1:687470/301‑1 (MQ=255)`  
                                                                                                                                                                                                                                                                                                        `agtcgCACGACAGAAAGAAATTGACCGCGCTAAGGCCCGTAAAGAACGTCACGAGGCGCGCTTAGAGGCACGCAGATTCAAACGTCGCAACCGCAAGGCACGTAAAGCACACAAAGCTAAGCGCGAAAGAATGCTTGCTGCGTGGCGATGGGCTGAACGTCAAGAACGGCGTAACCATGAGGTAGCTGTAGATGTACTAGGAAGAACCAATAACGCTATGCTCTGGGTCAACATGTTCTCTGGGGACTTTAAGGCGCTTGAGGAACGAATCGCGCTGCACTGGCGTAATGCTGACCAGATg  <  1:308256/301‑1 (MQ=255)`  

                                                                                                                                                                                                                                                                                                        `|`                                                                                                                                                                                                                                                                                                              
`TGCGAGCGTTTCAACAATGATGCCCATTATAAATACGCTGAGATTGCAAGCGACATCATTGATTGCAATTCGGATGAGCATGATGAGTTAACTGGATGGGATGGTGAGTTTGTTGAAACTTGTAAACTAATCCGCAAGTTCTTTGAGGGCATCGCCTCATTCGACATGCATAGCGGGAACATCATGTTCTCAAATGGAGACGTACCATACATCACCGACCCGGTATCATTCTCGCAGAAGAAAGACGGTGGCGCATTCAGCATCGACCCTGAGGAACTCATCAAGGAAGTCGAGGAAGTCGCACGACAGAAAGAAATTGACCGCGCTAAGGCCCGTAAAGAACGTCACGAGGGGCGCTTAGAGGCACGCAGATTCAAACGTCGCAACCGCAAGGCACGTAAAGCACACAAAGCTAAGCGCGAAAGAATGCTTGCTGCGTGGCGATGGGCTGAACGTCAAGAACGGCGTAACCATGAGGTAGCTGTAGATGTACTAGGAAGAACCAATAACGCTATGCTCTGGGTCAACATGTTCTCTGGGGACTTTAAGGCGCTTGAGGAACGAATCGCGCTGCACTGGCGTAATGCTGACCGGATG  >  NC_001604.1/2441‑3037`

  

Alignment Legend

Aligned base mismatch/match (shaded by quality score): `ATCG/ATCG` `< 3 ≤` `ATCG/ATCG` `< 11 ≤` `ATCG/ATCG` `< 24 ≤` `ATCG/ATCG` `< 36 ≤` `ATCG/ATCG` `< 38 ≤` `ATCG/ATCG`

Unaligned base: `atcg`    Masked matching base: `atcg`    Alignment gap: `‑`    Deleted base: `‑`
